# Role of emotional intelligence in shaping self-esteem among Indian nurses at Trichy, Tamilnadu

**DOI:** 10.6026/9732063002001564

**Published:** 2024-11-30

**Authors:** Rajathi A, Christena P, Rose Infantina J, Anitha Catherine M

**Affiliations:** 1 Department of Mental Health Nursing, KMC College of Nursing, Trichy, Tamilnadu, India; 2 Department of Obstetrics and Gynecological Nursing, KMC College of Nursing, Trichy, Tamilnadu, India; 3 Department of Mental Health Nursing, KMC College of Nursing, Trichy, Tamilnadu, India; 4 Department of Child Health Nursing, KMC College of Nursing, Trichy, Tamilnadu, India

**Keywords:** Emotional intelligence (EI), self-esteem, nurses

## Abstract

Nursing is a multifaceted profession that requires a blend of technical skills and emotional intelligence (EI) to manage various responsibilities, from patient care to team supervision. EI is closely connected to self-esteem, a psychological construct representing an individual's self-worth and competence. Self-esteem significantly influences how nurses perceive their abilities and engage with their work environment. The current study adopted the non-experimental descriptive correlational survey design to assess the emotional intelligence and self-esteem among the nurses. The study was conducted for a period of two months, among 301 Nurses working in various departments of the hospital, Trichy, Tamilnadu. The assessment of the samples was done using the background variables, TEIQue-SF- (Trait Emotional Intelligence questionnaire- short form) and Rosenberg's self-esteem scale. The data was collected through the Google Form for 4 weeks. The study results show that 81.1% of the nurses had average emotional intelligence only, 18.9% of the nurses had a high level of emotional intelligence and none of the nurses had a low level of emotional intelligence. In the aspects of self-esteem 76.4% of nurses had moderate level of self-esteem, 21.3% had high level of self-esteem and 2.3% had low level of self-esteem, there was a weak positive correlation between the level of Emotional intelligence and the level of Self- esteem with "r" value 0.321 & "p" value 0.01. Nurses render care to those who are emotionally heightened for patients and families. The persons exhibit confidence when they are emotionally intelligent, capable of understanding and stabilize their emotions. Self-esteem can be shaped by individuals' relationships with others, experiences and accomplishments in life.

## Background:

Nursing is a multifaceted profession that requires a blend of technical skills and emotional intelligence (EI) to manage various responsibilities, from patient care to team supervision [5]. EI is particularly significant in nursing as it encompasses the ability to perceive, understand and manage emotions-skills essential for personal and professional development. The term "Emotional Intelligence" was coined by Salovey and Mayer, who described it as the capacity to monitor self-emotion and other people emotions, guided in thinking and can modify ones' behaviors correspondingly [1]. In nursing, high EI is linked to better communication, problem-solving abilities and patient emotional support, which are critical for enhancing patient satisfaction and healthcare outcomes [2]. Emotional intelligence can be seen as a schedule that encircles the concept of emotional autonomy, coping and socially responsible behavior that predicts one's capacity and success to solve problems. It is a means through which we recognize, regulate, express and understand our emotions. Moreover, Emotional intelligence is closely connected to self-esteem, a psychological construct representing an individual's self-worth and competence [8]. EI is the ability to understand one's own feelings and to assess and respond to the feelings of others. It is linked to self-awareness, self-management, social awareness and social skills, all of which are vital in leadership roles [9]. Self-esteem significantly influences how nurses perceive their abilities and engage with their work environment. High self-esteem in nurses has been associated with positive professional behaviors such as better communication, enhanced sociability and overall job satisfaction [7]. It also contributes to resilience and adaptability in demanding healthcare settings, enabling nurses to cope with stress and maintain psychological health [4]. An individual's overall evaluation of self-worth, competence and suitability will be reflected by self-esteem and influence on holistic health. According to sociometer theory, self-esteem is an inner reflection of interpersonal relationships. If people are accepted and liked by others, their self-esteem rises and increases their positive emotions. When they get rejected, it reduces the self-esteem and results in negative emotions. Thus, mental well-being is not improved by self-esteem but can be improved by self- perception and acceptance. This further explains why social support, including organizational support, can enhance individual self-esteem [14, 16]. Thus, this study aims to explore the relationship between EI and self-esteem among nurses in Tamil Nadu, India, to provide insights that could inform targeted interventions for enhancing both personal well-being and professional effectiveness. By examining the levels of EI and self-esteem among nurses and their interrelationships, this study seeks to contribute to the broader discourse on nursing competencies and healthcare quality. Understanding these psychological constructs is vital for developing strategies that not only improve individual nurse outcomes but also enhance patient care delivery in diverse healthcare settings [6]. The research highlights on the title in assessing the role of emotional intelligence in shaping self-esteem among nurses at Trichy, Tamil Nadu Hospitals. The objectives of this study include, to assess the levels of Emotional Intelligence and Self-Esteem among nurses, to explore the relationship between emotional intelligence and self-esteem among nurses, to find out the association between the level of Emotional Intelligence and selected background variables among nurses and to find out the association between the level of Self-Esteem and selected background variables among nurses.

## Materials and Methods:

The current study adopted the non-experimental descriptive correlational survey design to assess the emotional intelligence and self-esteem among the nurses. The standardized tool was used after consultation with the subject experts. The data was collected through Google Forms from the nurses working in Hospitals of Trichy, Tamilnadu. The study was conducted for two months with nurses working in various departments of the hospital, aged between 20 and 60 years, qualified with the Diploma, Under-Graduated and Post-Graduated in Nursing were included in the study. Nurses who did not have Professional qualifications worked in the outpatient departments and had not given consent were excluded from the study. The pilot study was conducted for a week among 30 nurses to assess the feasibility of the tools prepared. The sample for the present study included 301 nurses working in various departments of the hospital. Based on the criteria mentioned above the non-probability purposive sampling technique was used to select the sample. The collected data were entered and coded in MS Excel. All the statistical analysis was done using IBM-SPSS 23 software. The data was tested statistically by Frequency distribution, Mean, Standard deviation, test for Level of significance and correlation.

## Study procedures:

Nurses working in Hospitals at Trichy, Tamilnadu who met the inclusion criteria were given detailed explanations about the study and tools. The samples were gathered in each hospital and the purpose of the study and questions was explained to the samples. Oral consent was obtained and confidentiality of the sample was maintained. The study period includes 08 June 2024 to 18 August 2024. The assessment of the samples includes totally 3 sections, first section is about the background variables (demographic variables, professional variables, work environmental variables & health variables), second is the assessment of the level of Emotional Intelligence using a standardized tool - TEIQue-SF- (Trait Emotional Intelligence questionnaire- short form) and the third section is the assessment of the level of Self-Esteem using a standardized tool - Rosenberg self-esteem scale. The data were collected through the Google-Form for 4 weeks.

## Development and description of the tools:

The Assessment of the samples was done with the tool development and includes totally 3 sections,

[1] The first section is about the background variables and consists of a total of 19 questions (demographic variables-8 questions, professional variables-4 questions, work environmental variables-4 questions & health variables-3 questions).

[2] The second is the assessment of the level of Emotional Intelligence using a standardized tool - TEIQue-SF- (Trait Emotional Intelligence questionnaire- short form), it is a scale with the 07 possible responses for each statement, each statement is rated on a scale of 1-7 (1=completely disagree, 2= disagree, 3= somewhat disagree, 4= neither agree nor disagree, 5= somewhat agree, 6= agree, 7=completely agree), average items 1-30. Items 2,4,5,7,8,10,12,13,14,16,18,22,25,26 & 28 are reverse scored.

[3] The higher scores on these measures indicate a higher level of emotional intelligence [11].

[4] The third section is the assessment of the level of Self-Esteem using a standardized tool - Rosenberg self-esteem scale, it consists of 10 statements that respondent's rate on a 4-point scale ranging from strongly agree to strongly disagree, the positive items (1, 3, 4, 6, 8) are scored 3- strongly agree to 0-strongly disagree & the negative items (2, 5, 7, 9 & 10) are reverse scored 0-strongly agree to 3-strongly disagree. The total score ranges from 0-30, with higher scores indicating higher self-esteem [12, 15].

## Results:

**Figure 1:**
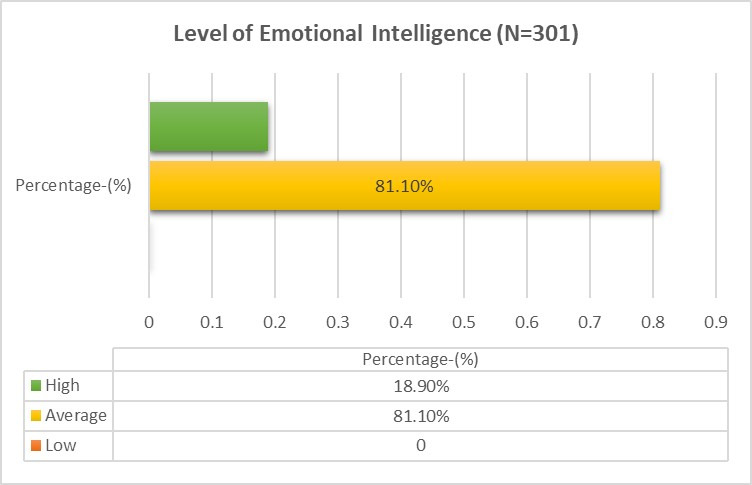
Displays the overall level of Emotional Intelligence and results that 81.1% of the nurses had average emotional intelligence and 18.9% of the nurses had high level of emotional intelligence among the Nurses working in Hospitals at Trichy, Tamilnadu

**Figure 2:**
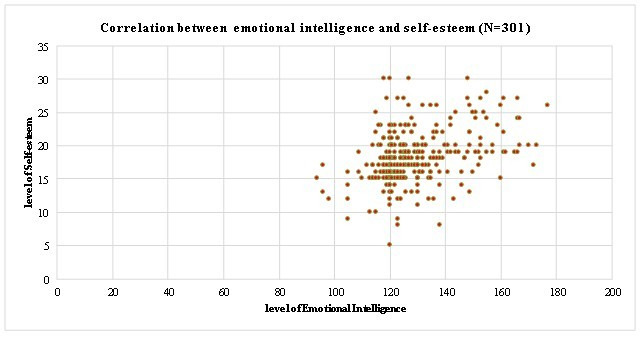
Displays the overall level of Self- Esteem and results as 76.4%of the nurses had moderate level of self-esteem, 21.3% of the nurses had high level of self-esteem and 2.3% had low level of self-esteem among the Nurses working in Hospitals at Trichy, Tamilnadu

**Figure 3:**
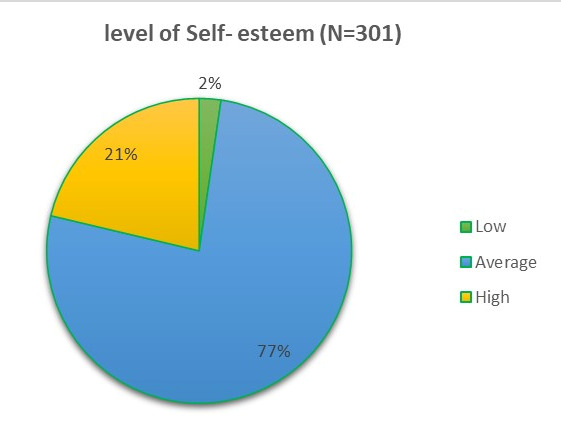
Displays there is weak positive Correlation between emotional intelligence and self-esteem among the Nurses working in Hospitals at Trichy, Tamilnadu

A total of 321 nurse samples are recorded. In which, 20 nurses were excluded as they did not meet the inclusion criteria and the 301 nurses were analyzed for the study. The background variables of nurses, shows that 86.7 % of the nurses belongs to 20-30 years of age, female were 97.3% , 77.1 % were single, 49.5% completed Bachelors in Nursing , 49.5% belongs to rural area , 68.8% from the nuclear family, 67.1% them earn Rs.10000-15000, 55.8% staying at hostel, 71.8% nurses had 1-3 Years of experience, 80.7% nurses having 8 hours shift, 97.7% hold the past experience exposure within Tamilnadu, 48.8% nurses had tenure stability of 1-3 years, 70.8% with moderate workload, 57.5% satisfied with the job, 70.1% of nurses experiences moderate stress level, 51.2% of nurses had moderate support system, 74.1 % had good physical health, 71.4% with good mental health status and 64.5% had good sleep quality. Most of the nurses (81.1%) had average emotional intelligence and (18.9%) had high level of emotional intelligence and nurses had no low level of emotional intelligence (Figure 1). Most of the nurses (76.4%) had moderate level of self-esteem, (21.3%) had high level of self-esteem and (2.3%) had low level of self-esteem (Figure 2). There was a weak positive correlation between the level of emotional intelligence and the level of Self- esteem with "r" value-0.321 & "p" value-0.01 (Figure 3).

Table 1 show that the mean value is 129.09 & SD is 14.308 for Emotional intelligence and the mean value is 18.17 & SD is 4.015 for Self-esteem. Table 2 states there is a significant association between the level of emotional intelligence and selected background variables (marital status, education qualification, place of stay, years of experience, past experience exposure, tenure stability, workload, job satisfaction, stress levels, physical health status and mental health status and sleep quality). Table 3 shows there is a significant association between the level of self-esteem and selected background variables (age, education qualification, income and years of experience, job satisfaction, Support system, physical health status and mental health status and sleep quality). Table 4 presents, there was a weak positive correlation between the level of Emotional intelligence and the level of Self- esteem, "r" value-0.321 & "p" value-0.01.

Table 1 results as 129.1 (Mean) and 14.308 (SD) for Emotional intelligence whereas 18.17 (Mean) and 04.015 (SD) for Self- esteem. Table 2 results that there is a significant association between the level of emotional intelligence and selected background variables (marital status, education qualification, place of stay, years of experience, past experience exposure, tenure stability, workload, job satisfaction. stress levels, physical health status, mental health status and sleep quality). Table 3 results that there is a significant association between the level of self-esteem and selected background variables (age, education qualification, income, years of experience, job satisfaction, Support system, physical health status and mental health status and sleep quality. Table 4 showed there was a weak positive correlation between the level of emotional intelligence and the level of Self- esteem with "r" value-0.321 & "p" value-0.01.

## Discussion:

The study suggest that emotions play an remarkable role in the field of the nursing profession which is in need of the professional expertise and psychological based care, broader knowledge about the self and emotions in nursing would be very important for the future professional development. The capability of managing self-emotions and understands other's is especially useful in the practice of this profession successfully. A nurse with the sound emotional intelligence can work with his/her thoughts and feelings in balance. In the workplace, Emotional intelligence is considered to play a significant role. It is a prerequisite for all profession that dealt with the human relations like nursing [3].

Tripathi conducted a quantitative study to investigate the relationship between self- esteem and emotional intelligence and marital satisfaction among women in Karaj in Iran. Study was conducted with the sample of 100 women with simple random sampling framework [13]. It was found positive relationship exists between the self- esteem and emotional intelligence and marital satisfaction among women in Kuraj in Iran.

## The objectives of this study:

[1] To assess the Levels of Emotional Intelligence and Self-Esteem among Nurses in Tamil Nadu Hospitals.

The study reveals that 81.1% of the nurses had average emotional intelligence and 18.9% of the nurses had high level of emotional intelligence whereas 76.4%of the nurses had moderate level of self-esteem, 21.3% of the nurses had high level of self-esteem and 2.3% had low level of self-esteem

[2] To find out the association between the level of Emotional Intelligence and selected Variables among nurses and to find out the association between the level of Self-Esteem and selected Variables among nurses.

The study shows that there is a significant association between the level of emotional intelligence and selected background variables (marital status, education qualification, place of stay, years of experience, past experience exposure, tenure stability, workload, job satisfaction., stress levels, physical health status, mental health status and sleep quality) and there is a significant association between the level of self-esteem and selected background variables (age, education qualification, income, years of experience, job satisfaction, Support system, physical health status, mental health status and sleep quality.

[3] To explore the relationship between emotional intelligence and self-esteem among nurses.

The study stating that there was a weak positive correlation between the level of emotional intelligence and the level of Self-esteem with "r" value 0.321 & "p" value 0.01

## Conclusion:

Nurses render care to those who are emotionally delicate for patients and families. The persons exhibit confidence when they are emotionally intelligent, capable of understanding and stabilize their emotions. Self-esteem can be shaped by individuals' relationships with others, experiences and accomplishments in life. Effective emotional control increases one's self-esteem which in turn enables people to be more adaptive to external stressors with greater psychological well-being [10]. Thus, a positive relationship between emotional intelligence and self-esteem, findings indicated a weak positive relationship between emotional intelligence and self-esteem.

## Recommendation:

This study will serve as a basis for nurses and nurse administrators to gain a root-level understanding that there can be a relationship between emotional intelligence and self-esteem, wherein different interventions can be made for the nurses to enhance the levels of positive assertiveness, stress management, emotional regulation, positive self-talk, team building and communication strategies.

## Figures and Tables

**Table 1:** Mean, standard deviation of Emotional intelligence and Self- esteem among staff nurses (N=301)

**Variables**	**Maximum Score**	**Mean**	**Standard Deviation**
Emotional intelligence	210	129.1	14.308
Self- esteem	30	18.17	4.015

**Table 2:** Association between level of emotional intelligence and selected variables among staff nurses (N=301)

**Variable**	**Chi-square test**	**df**	**P- value**
**Demographic Variable**			
Marital status	15.4	2	*0.000
Educational Qualification	8.6	2	*0.13
Place of stay	6.8	1	*0.009
Professional variables			
Years of Experience	39.5	4	*0.000
Work Shift	6.9	3	*0.074
Experience exposure	20.8	1	*0.000
Tenure Stability	25.4	4	*0.000
Work Environment variables			
Work Load	5.2	4	*0.075
Job satisfaction	12	4	*0.018
Stress levels	7.9	2	*0.019
Health variables			
Physical Health status	10.4	3	*0.016
Mental Health status	6	3	*0.114
Sleep Quality	5.3	3	*0.146

**Table 3:** Association between level of self- esteem and selected variables among staff nurses (N=301)

**Variable**	**Chi-square test**	**df**	**P- value**
**Demographic Variable**			
Age	6.9	3	*0.32
Educational qualification	14.53	4	*0.006
Income	9.489	6	*0.148
**Professional variables**			
Years of Experience	12.967	8	*0.113
**Work Environment variables**			
Job satisfaction	13.553	8	*0.094
Support system	7.204	4	*0.125
**(family/friends/colleagues)**			
**Health variables**			
Physical Health status	15.196	6	*0.019
Mental Health status	18.193	6	*0.006
Sleep Quality	24.442	6	*0.000

**Table 4:** Correlation between emotional intelligence and self-esteem (N=301)

**Variables**	**"r" value**	**"p" value**	**correlation**
Emotional intelligence	0.321	0.01	Weak positive
Self- esteem			
